# Expected net gain data of low-template DNA analyses

**DOI:** 10.1016/j.dib.2016.05.059

**Published:** 2016-05-30

**Authors:** Simone Gittelson, Carolyn R. Steffen, Michael D. Coble

**Affiliations:** National Institute of Standards and Technology, 100 Bureau Drive, Gaithersburg, MD 20899, United States

**Keywords:** Forensic science, LT-DNA, Replicates

## Abstract

Low-template DNA analyses are affected by stochastic effects which can produce a configuration of peaks in the electropherogram (EPG) that is different from the genotype of the DNA׳s donor. A probabilistic and decision-theoretic model can quantify the expected net gain (ENG) of performing a DNA analysis by the difference between the expected value of information (EVOI) and the cost of performing the analysis. This article presents data on the ENG of performing DNA analyses of low-template DNA for a single amplification, two replicate amplifications, and for a second replicate amplification given the result of a first analysis. The data were obtained using amplification kits AmpF*l*STR Identifiler Plus and Promega׳s PowerPlex 16 HS, an ABI 3130xl genetic sequencer, and Applied Biosystem׳s GeneMapper ID-X software. These data are supplementary to an original research article investigating whether a forensic DNA analyst should perform a single DNA analysis or two replicate analyses from a decision-theoretic point of view, entitled “Low-template DNA: a single DNA analysis or two replicates?” (Gittelson et al., 2016) [1].

**Specifications Table**

**Table:** 

Subject area	Biology
More specific subject area	Forensic science
Type of data	Table, Graph
How data was acquired	Amplification kits AmpF*l*STR Identifiler Plus (29 cycles) and Promega׳s PowerPlex 16 HS (32 cycles), capillary electrophoresis, ABI 3130xl genetic sequencer (default injection settings), Applied Biosystems׳ GeneMapper ID-X software version 1.3
Data format	Analyzed
Experimental factors	Dilution of DNA samples to 10 pg/μL, 7.5 pg/μL, 5 pg/μL, 2.5 pg/μL, 1 pg/μL, 0.75 pg/μL, 0.5 pg/μL, and 0.25 pg/μL. DNA analysis was performed on 1 μL.
Experimental features	Electropherograms were obtained for a range of low-level DNA quantities. The expected net gain of the DNA results was quantified based on a probabilistic and decision-theoretic model.
Data source location	Gaithersburg, MD, United States of America
Data accessibility	Data are in this article.

**Value of the data**

• Forensic genetic laboratories can use this data to make rational decisions about replicate DNA analyses of low-template DNA.
• The forensic science community can use this data to develop low-template DNA analysis guidelines and protocols.
• Researchers can compare this data with the expected net gain (ENG) of other DNA analysis methods.

## 1. Data

This dataset consists of graphs that present the ENG of low-template DNA analyses in function of the average allelic peak height for a set of different parameter values covering the amplification kit, the probability of allele drop-in, the utility function and the DNA analysis costs.

Fig. 1, Fig. 2, Fig. 3, Fig. 4, Fig. 5, Fig. 6, Fig. 7, Fig. 8 present the ENG of concentrating the DNA extract in a single amplification and the ENG of splitting the extract into two amplification tubes to produce two replicates. We call this the *all in vs. two replicates* data.

Fig. 9, Fig. 10, Fig. 11, Fig. 12, Fig. 13, Fig. 14, Fig. 15, Fig. 16 present the ENG of performing a DNA analysis to obtain a second replicate in a case where an electropherogram (EPG) has already been obtained from a first analysis. We call this the *additional replicate* data.

## 2 Experimental design, materials and methods

### 2.1 DNA analyses

#### 2.1.1 DNA samples

Single-source DNA dilution samples were prepared from the DNA of two donors who are heterozygous at each of the target loci of the amplification kits AmpF*l*STR Identifiler Plus and Promega׳s PowerPlex 16 HS. The dilutions were prepared from a master mix to create DNA samples of the following concentrations: 10 pg/μL, 7.5 pg/μL, 5 pg/μL, 2.5 pg/μL, 1 pg/μL, 0.75 pg/μL, 0.5 pg/μL, and 0.25 pg/μL. For each DNA analysis, 1 μL was taken from these solutions, producing EPGs for DNA quantities of 10 pg, 7.5 pg, 5 pg, 2.5 pg, 1 pg, 0.75 pg, 0.5 pg and 0.25 pg. This range was chosen because it created EPGs ranging from having no allele or locus drop-outs to showing all loci dropping out for an analytical threshold of 10 rfu.

Two datasets were collected: the first consisted of 10 replicates for each quantity and for each donor, and the second consisted of 10 replicates for 2.5 pg and 0.25 pg and 20 replicates for 1 pg, 0.75 pg and 0.5 pg for each donor. The purpose of the second dataset was to obtain more data for the DNA quantities that were necessary for determining the parameter values for the model that assigns the probability of allele drop-out (see Section 2.2.2).

#### 2.1.2 PCR amplification and detection

Two kits were used for the DNA amplification: AmpF*l*STR Identifiler Plus (29 cycles) and PowerPlex 16 HS by Promega (32 cycles). The cycle numbers correspond to the manufacturers׳ recommendations for low DNA amounts. Capillary electrophoresis separated and detected the PCR products on am ABI 3130xl genetic sequencer. The injection settings were the default settings of 10 s at 3 kV and 5 s at 3 kV for Identifiler Plus and PowerPlex 16 HS, respectively.

#### 2.1.3 Analysis of typing results

GeneMapper ID-X software version 1.3 by Applied Biosystems was used for analysing the DNA typing results. To determine the parameter values for the probability of allele drop-out model, the analytical threshold was set to 10 rfu and all artefact and stutter peaks were removed.

### 2.2 Probabilistic model

The probabilities required for the decision analysis were assigned using a semi-continuous model. This model did not take into account the presence of non-allelic signals (*e.g.*, stutters, analytical artefacts). It considered the results at each locus to be conditionally independent of the results at the other loci given the model׳s parameter values. We used the *R* software^1^ to perform the probabilistic computations according to the equations presented in [2] and the specificities described below.

#### 2.2.1 Allele probabilities

Allele probabilities were assigned as point estimates:

$$p_{A}=\frac{n_{A}+\frac{1}{k}}{N+1},$$

where *p*_*A*_ denotes the allele probability for allele *A*, *n*_*A*_ is the number of *A* alleles observed, *k* is the number of unique allele designations that have been observed for that locus, and *N* is the total number of observed alleles for that locus. The allele probabilities in this model are based on the allele frequency data published in [3].

#### 2.2.2 Probability of allele drop-out

The probability of allele drop-out, Pr(D), was assigned as [4]:

(1) $$\mathrm{Pr}(D)=\frac{e^{\beta_{0}+\beta_{1}\;\ln(\hat{H})}}{1+e^{\beta_{0}+\beta_{1}\;\ln(\hat{H})}},$$

with $\beta_{0}=6.3244$ and $\beta_{1}=-1.6632$ for Identifiler Plus and $\beta_{0}=6.6044$ and $\beta_{1}=-1.7360$ for PowerPlex 16 HS. Table 1 presents the parameter values obtained for each donor, dataset and kit.

#### 2.2.3 Probability of allele drop-in

This model assumes that there is at most one drop-in allele per locus and models the probability of allele drop-in as a constant. To take into account the range of possible values, we performed two sets of decision analyses: one for a probability of allele drop-in of 0.01 per locus, and one for a probability of allele drop-in of 0.05 per locus.

### 2.3 Decision-theoretic model

The designation of the donor׳s genotype was modelled with a decision-theoretic model [5], and the ENG was quantified as the difference between the expected value of information (EVOI) and the cost of performing the analysis. The EVOI was quantified using the approach explained in [1]. Decision analyses were performed for a symmetric and a conservative preference structure, and for a range of magnitudes. Table 1 in [1] presents these preference structures and the definition of *m*, which is used for defining the utility function׳s magnitude. For further explanations on the utility function and the quantification of the EVOI of a DNA analysis, we refer the reader to [1].

## Disclaimer

Certain commercial equipment, instruments, and suppliers are identified in this paper to foster understanding. Such identification does not imply recommendation or endorsement by the National Institute of Standards and Technology, nor does it imply that the materials or equipment identified are necessarily the best available for the purpose.

## Figures and Tables

**Fig. 1:**
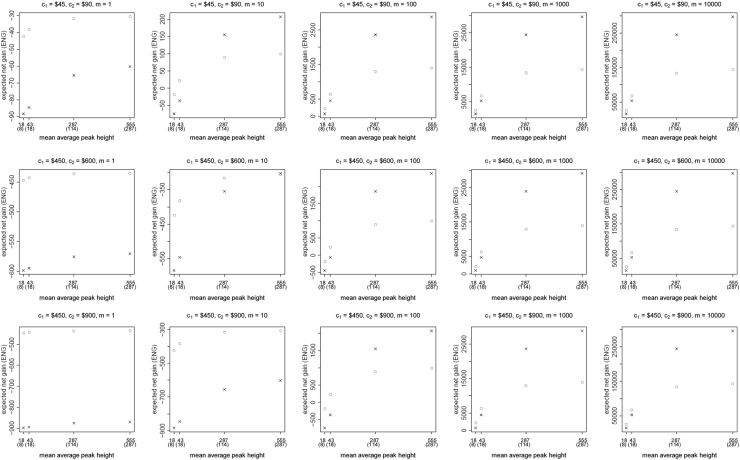
The Identifiler Plus *all in vs. two replicates* data for a symmetric preference structure and a probability of allele drop-in of 0.01. These graphs show the ENGs of a single DNA analysis (○) and of two replicates (×) in function of the mean average allelic peak height in an EPG. The value outside the brackets is the mean average peak height (in rfu) for a single analysis and the value in brackets the mean average allelic peak height (in rfu) in each of the two replicates. From left to right, the graphs show the results for increasing values of the utility function׳s magnitude, *m*, for values of *m* equal to 1, 10, 100, 1000 and 10,000. The first row of graphs presents the results for DNA analysis costs of $45 for one analysis and $90 for two replicates, the second row for costs of $450 for one analysis and $600 for two replicates, and the third row for costs of $450 for one analysis and $900 for two replicates.

**Fig. 2:**
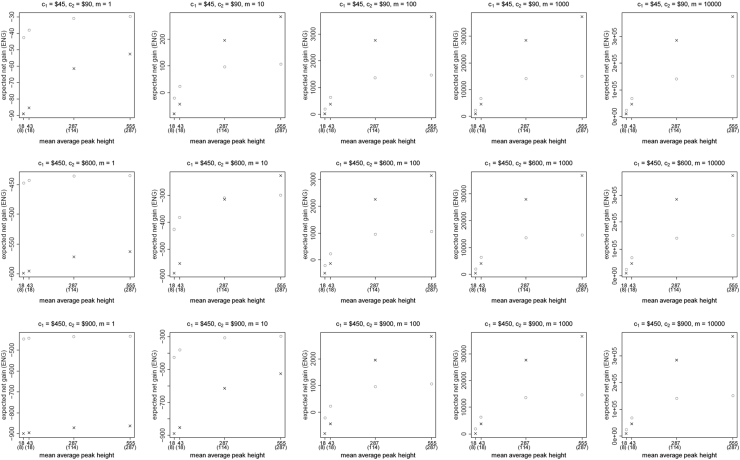
The Identifiler Plus *all in vs. two replicates* data for a symmetric preference structure and a probability of allele drop-in of 0.05. These graphs show the ENGs of a single DNA analysis (○) and of two replicates (×) in function of the mean average allelic peak height in an EPG. The value outside the brackets is the mean average peak height (in rfu) for a single analysis and the value in brackets the mean average allelic peak height (in rfu) in each of the two replicates. From left to right, the graphs show the results for increasing values of the utility function׳s magnitude, *m*, for values of *m* equal to 1, 10, 100, 1000 and 10,000. The first row of graphs presents the results for DNA analysis costs of $45 for one analysis and $90 for two replicates, the second row for costs of $450 for one analysis and $600 for two replicates, and the third row for costs of $450 for one analysis and $900 for two replicates.

**Fig. 3:**
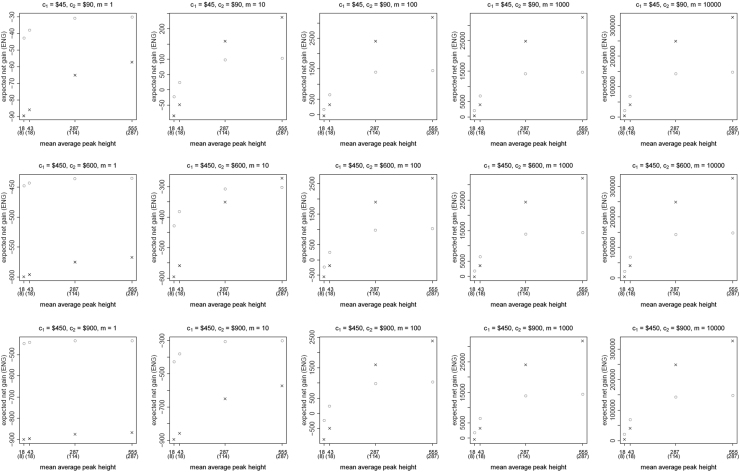
The Identifiler Plus *all in vs. two replicates* data for a conservative preference structure and a probability of allele drop-in of 0.01. These graphs show the ENGs of a single DNA analysis (○) and of two replicates (×) in function of the mean average allelic peak height in an EPG. The value outside the brackets is the mean average peak height (in rfu) for a single analysis and the value in brackets the mean average allelic peak height (in rfu) in each of the two replicates. From left to right, the graphs show the results for increasing values of the utility function׳s magnitude, *m*, for values of *m* equal to 1, 10, 100, 1000 and 10,000. The first row of graphs presents the results for DNA analysis costs of $45 for one analysis and $90 for two replicates, the second row for costs of $450 for one analysis and $600 for two replicates, and the third row for costs of $450 for one analysis and $900 for two replicates.

**Fig. 4:**
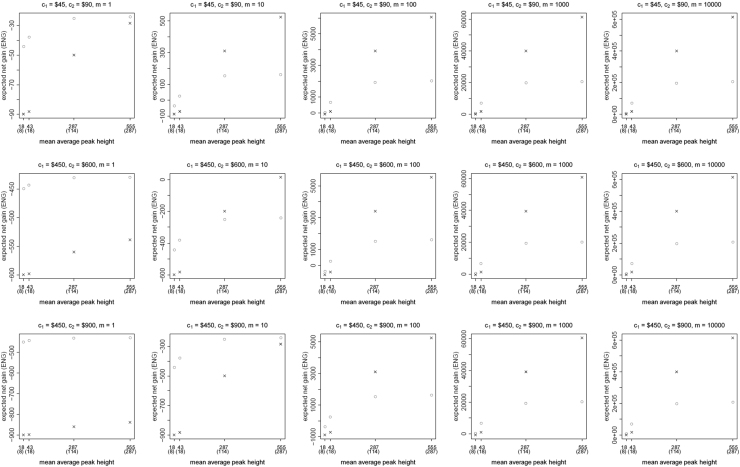
The Identifiler Plus *all in vs. two replicates* data for a conservative preference structure and a probability of allele drop-in of 0.05. These graphs show the ENGs of a single DNA analysis (○) and of two replicates (×) in function of the mean average allelic peak height in an EPG. The value outside the brackets is the mean average peak height (in rfu) for a single analysis and the value in brackets the mean average allelic peak height (in rfu) in each of the two replicates. From left to right, the graphs show the results for increasing values of the utility function׳s magnitude, *m*, for values of *m* equal to 1, 10, 100, 1000 and 10,000. The first row of graphs presents the results for DNA analysis costs of $45 for one analysis and $90 for two replicates, the second row for costs of $450 for one analysis and $600 for two replicates, and the third row for costs of $450 for one analysis and $900 for two replicates.

**Fig. 5:**
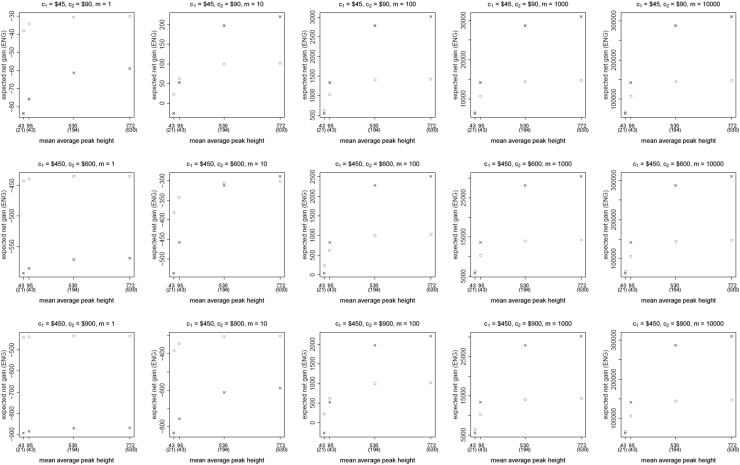
The PowerPlex 16 HS *all in vs. two replicates* data for a symmetric preference structure and a probability of allele drop-in of 0.01. These graphs show the ENGs of a single DNA analysis (○) and of two replicates (×) in function of the mean average allelic peak height in an EPG. The value outside the brackets is the mean average peak height (in rfu) for a single analysis and the value in brackets the mean average allelic peak height (in rfu) in each of the two replicates. From left to right, the graphs show the results for increasing values of the utility function׳s magnitude, *m*, for values of *m* equal to 1, 10, 100, 1000 and 10,000. The first row of graphs presents the results for DNA analysis costs of $45 for one analysis and $90 for two replicates, the second row for costs of $450 for one analysis and $600 for two replicates, and the third row for costs of $450 for one analysis and $900 for two replicates.

**Fig. 6:**
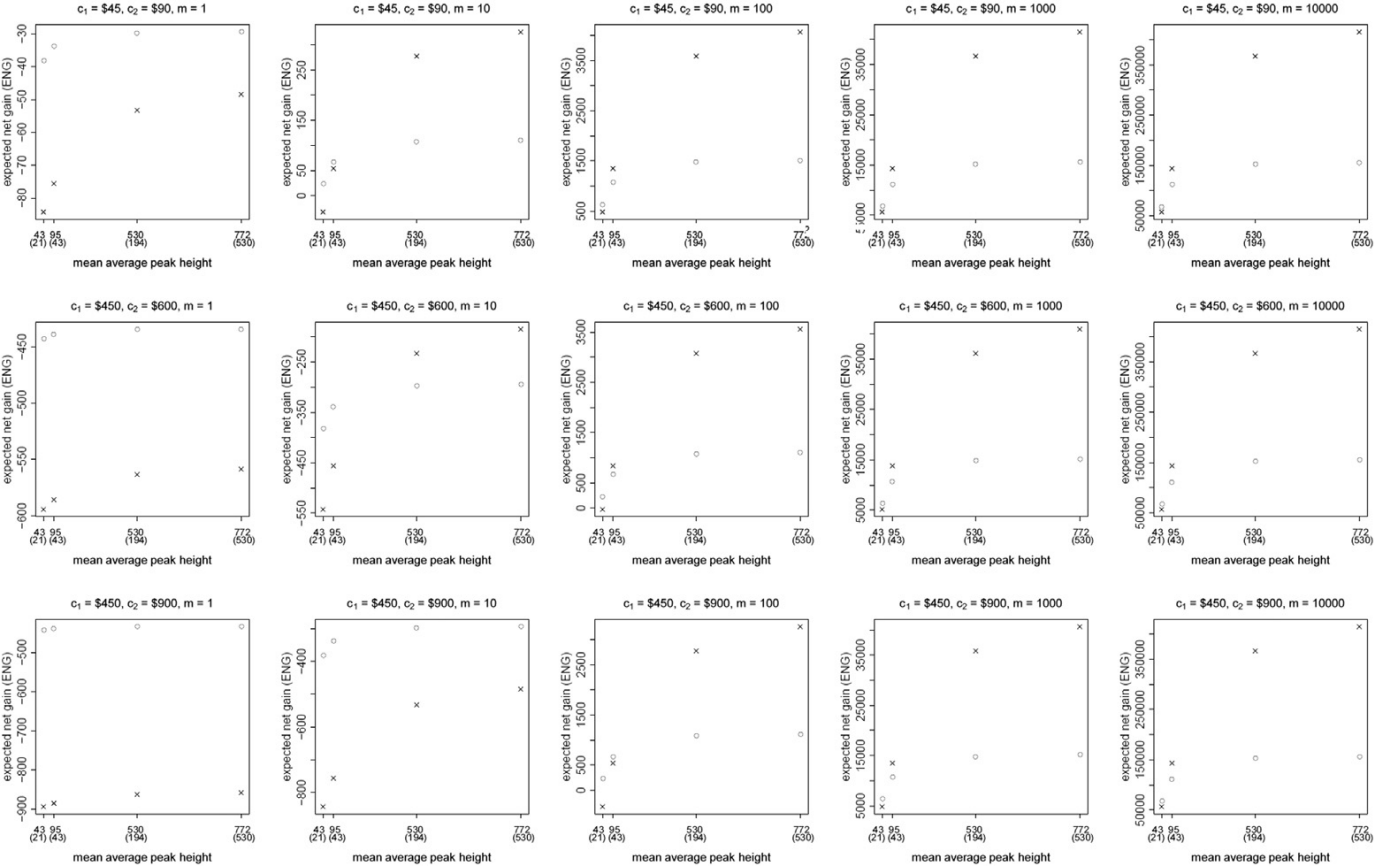
The PowerPlex 16 HS *all in vs. two replicates* data for a symmetric preference structure and a probability of allele drop-in of 0.05. These graphs show the ENGs of a single DNA analysis (○) and of two replicates (×) in function of the mean average allelic peak height in an EPG. The value outside the brackets is the mean average peak height (in rfu) for a single analysis and the value in brackets the mean average allelic peak height (in rfu) in each of the two replicates. From left to right, the graphs show the results for increasing values of the utility function׳s magnitude, *m*, for values of *m* equal to 1, 10, 100, 1000 and 10,000. The first row of graphs presents the results for DNA analysis costs of $45 for one analysis and $90 for two replicates, the second row for costs of $450 for one analysis and $600 for two replicates, and the third row for costs of $450 for one analysis and $900 for two replicates.

**Fig. 7:**
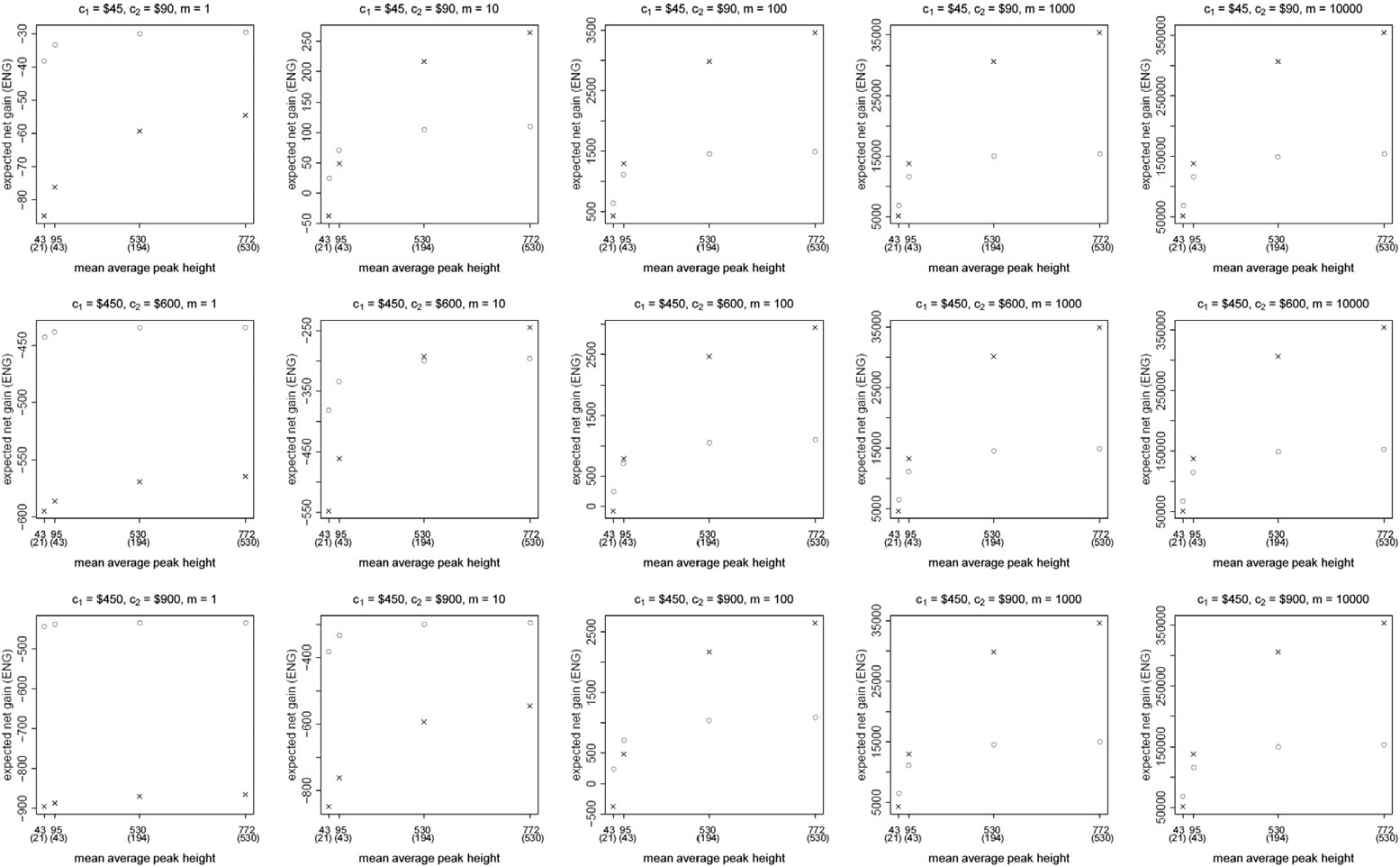
The PowerPlex 16 HS *all in vs. two replicates* data for a conservative preference structure and a probability of allele drop-in of 0.01. These graphs show the ENGs of a single DNA analysis (○) and of two replicates (×) in function of the mean average allelic peak height in an EPG. The value outside the brackets is the mean average peak height (in rfu) for a single analysis and the value in brackets the mean average allelic peak height (in rfu) in each of the two replicates. From left to right, the graphs show the results for increasing values of the utility function׳s magnitude, *m*, for values of *m* equal to 1, 10, 100, 1000 and 10,000. The first row of graphs presents the results for DNA analysis costs of $45 for one analysis and $90 for two replicates, the second row for costs of $450 for one analysis and $600 for two replicates, and the third row for costs of $450 for one analysis and $900 for two replicates.

**Fig. 8:**
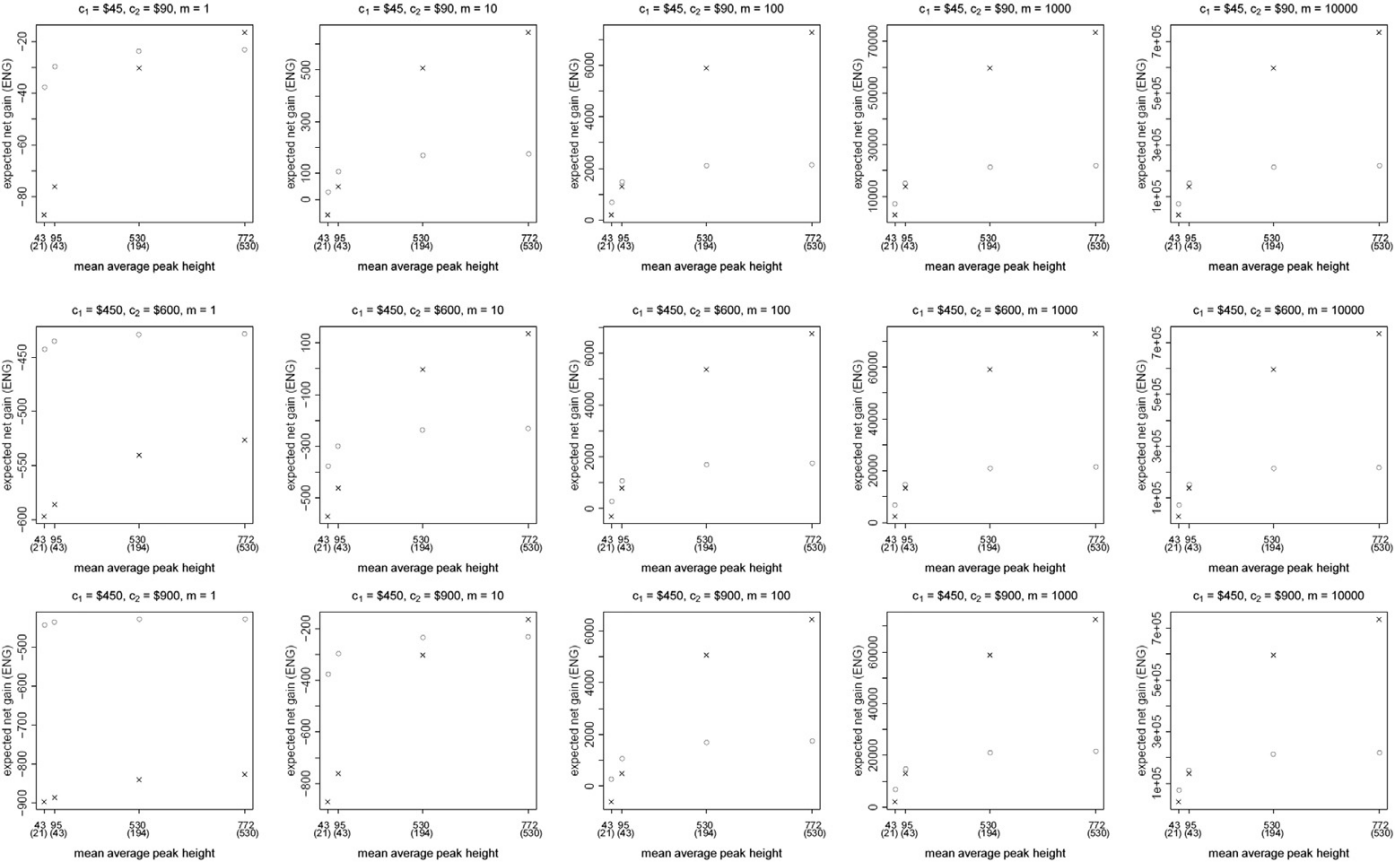
The PowerPlex 16 HS *all in vs. two replicates* data for a conservative preference structure and a probability of allele drop-in of 0.05. These graphs show the ENGs of a single DNA analysis (○) and of two replicates (×) in function of the mean average allelic peak height in an EPG. The value outside the brackets is the mean average peak height (in rfu) for a single analysis and the value in brackets the mean average allelic peak height (in rfu) in each of the two replicates. From left to right, the graphs show the results for increasing values of the utility function׳s magnitude, *m*, for values of *m* equal to 1, 10, 100, 1000 and 10,000. The first row of graphs presents the results for DNA analysis costs of $45 for one analysis and $90 for two replicates, the second row for costs of $450 for one analysis and $600 for two replicates, and the third row for costs of $450 for one analysis and $900 for two replicates.

**Fig. 9:**
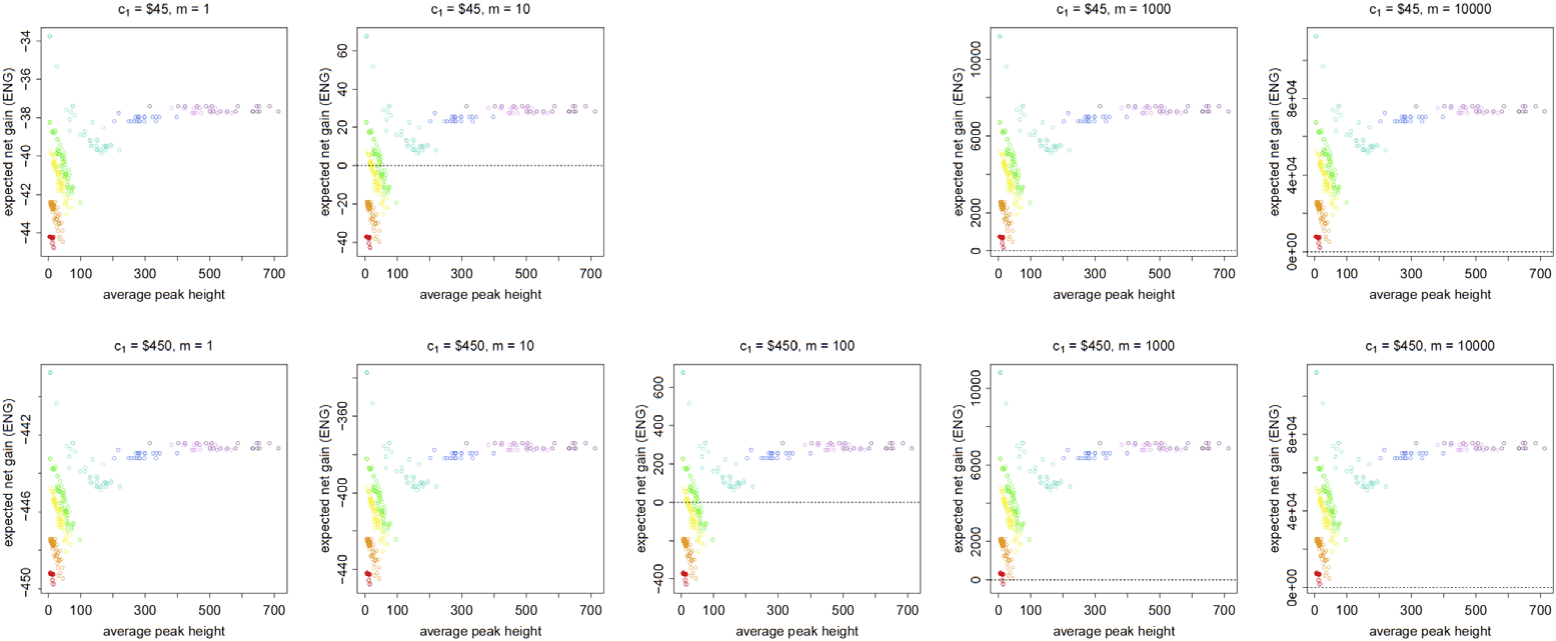
The Identifiler Plus *additional replicate* data for a symmetric preference structure and a probability of allele drop-in of 0.01. These graphs show the ENGs of a second replicate in function of the average allelic peak height (in rfu) of the first DNA analysis׳s EPG for DNA samples quantified as ≈0.25 pg (red), ≈0.5 pg (orange), ≈0.75 pg (yellow), ≈1 pg (green), ≈2.5 pg (turquoise), ≈5 pg (blue), ≈7.5 pg (light magenta) and ≈10 pg (dark magenta). From left to right, the graphs show the results for increasing values of the utility function׳s magnitude, *m*, for values of *m* equal to 1, 10, 100, 1000 and 10,000. The first row of graphs presents the results for a cost of $45 per DNA analysis, and the second row for a cost of $450 per DNA analysis. The graph for *m*=100 and a cost of $45 per DNA analysis is not presented here because it is published as Fig. 1 in [1].

**Fig. 10:**
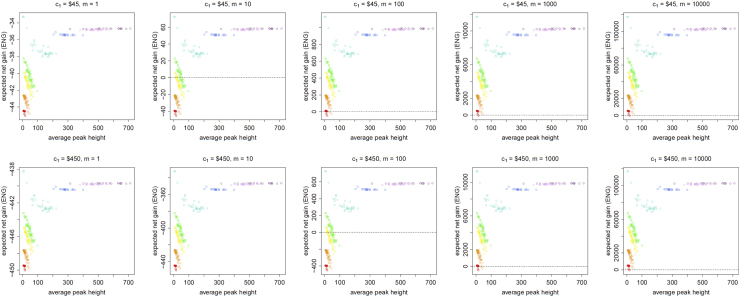
The Identifiler Plus *additional replicate* data for a symmetric preference structure and a probability of allele drop-in of 0.05. These graphs show the ENGs of a second replicate in function of the average allelic peak height (in rfu) of the first DNA analysis׳s EPG for DNA samples quantified as ≈0.25 pg (red), ≈0.5 pg (orange), ≈0.75 pg (yellow), ≈1 pg (green), ≈2.5 pg (turquoise), ≈5 pg (blue), ≈7.5 pg (light magenta) and ≈10 pg (dark magenta). From left to right, the graphs show the results for increasing values of the utility function׳s magnitude, *m*, for values of *m* equal to 1, 10, 100, 1000 and 10,000. The first row of graphs presents the results for a cost of $45 per DNA analysis, and the second row for a cost of $450 per DNA analysis.

**Fig. 11:**
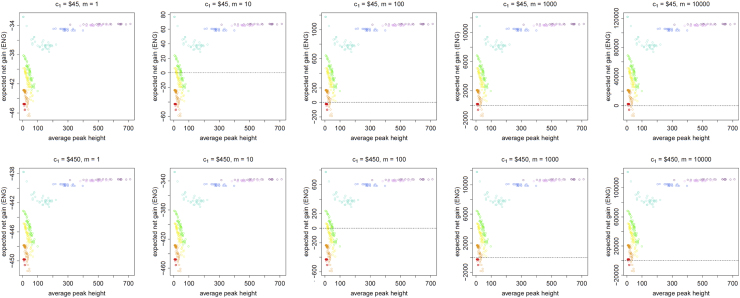
The Identifiler Plus *additional replicate* data for a conservative preference structure and a probability of allele drop-in of 0.01. These graphs show the ENGs of a second replicate in function of the average allelic peak height (in rfu) of the first DNA analysis׳s EPG for DNA samples quantified as ≈0.25 pg (red), ≈0.5 pg (orange), ≈0.75 pg (yellow), ≈1 pg (green), ≈2.5 pg (turquoise), ≈5 pg (blue), ≈7.5 pg (light magenta) and ≈10 pg (dark magenta). From left to right, the graphs show the results for increasing values of the utility function׳s magnitude, *m*, for values of *m* equal to 1, 10, 100, 1000 and 10,000. The first row of graphs presents the results for a cost of $45 per DNA analysis, and the second row for a cost of $450 per DNA analysis.

**Fig. 12:**
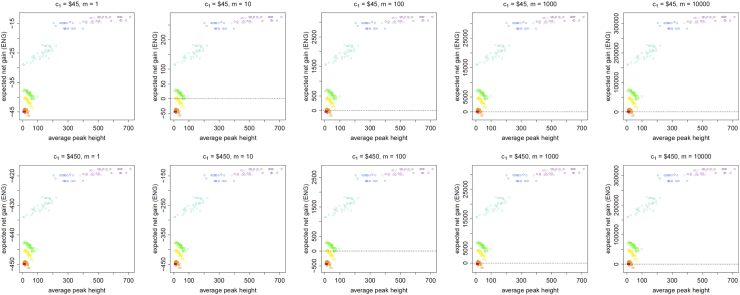
The Identifiler Plus *additional replicate* data for a conservative preference structure and a probability of allele drop-in of 0.05. These graphs show the ENGs of a second replicate in function of the average allelic peak height (in rfu) of the first DNA analysis׳s EPG for DNA samples quantified as ≈0.25 pg (red), ≈0.5 pg (orange), ≈0.75 pg (yellow), ≈1 pg (green), ≈2.5 pg (turquoise), ≈5 pg (blue), ≈7.5 pg (light magenta) and ≈10 pg (dark magenta). From left to right, the graphs show the results for increasing values of the utility function׳s magnitude, *m*, for values of *m* equal to 1, 10, 100, 1000 and 10,000. The first row of graphs presents the results for a cost of $45 per DNA analysis, and the second row for a cost of $450 per DNA analysis.

**Fig. 13:**
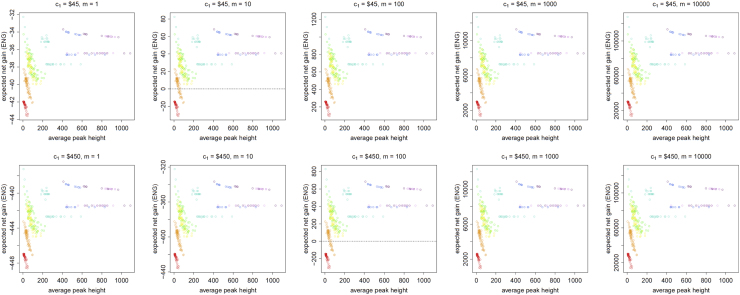
The PowerPlex 16 HS *additional replicate* data for a symmetric preference structure and a probability of allele drop-in of 0.01. These graphs show the ENGs of a second replicate in function of the average allelic peak height (in rfu) of the first DNA analysis׳s EPG for DNA samples quantified as ≈0.25 pg (red), ≈0.5 pg (orange), ≈0.75 pg (yellow), ≈1 pg (green), ≈2.5 pg (turquoise), ≈5 pg (blue), ≈7.5 pg (light magenta) and ≈10 pg (dark magenta). From left to right, the graphs show the results for increasing values of the utility function׳s magnitude, *m*, for values of *m* equal to 1, 10, 100, 1000 and 10,000. The first row of graphs presents the results for a cost of $45 per DNA analysis, and the second row for a cost of $450 per DNA analysis.

**Fig. 14:**
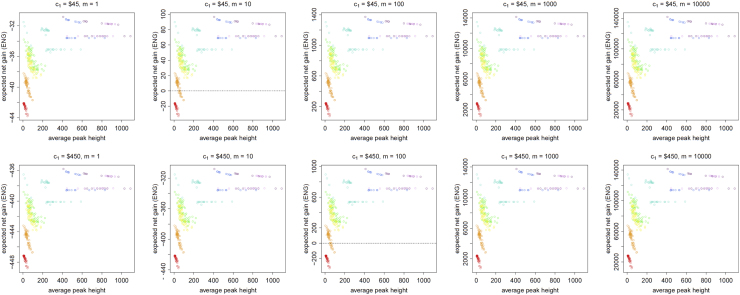
The PowerPlex 16 HS *additional replicate* data for a symmetric preference structure and a probability of allele drop-in of 0.05. These graphs show the ENGs of a second replicate in function of the average allelic peak height (in rfu) of the first DNA analysis׳s EPG for DNA samples quantified as ≈0.25 pg (red), ≈0.5 pg (orange), ≈0.75 pg (yellow), ≈1 pg (green), ≈2.5 pg (turquoise), ≈5 pg (blue), ≈7.5 pg (light magenta) and ≈10 pg (dark magenta). From left to right, the graphs show the results for increasing values of the utility function׳s magnitude, *m*, for values of *m* equal to 1, 10, 100, 1000 and 10,000. The first row of graphs presents the results for a cost of $45 per DNA analysis, and the second row for a cost of $450 per DNA analysis.

**Fig. 15:**
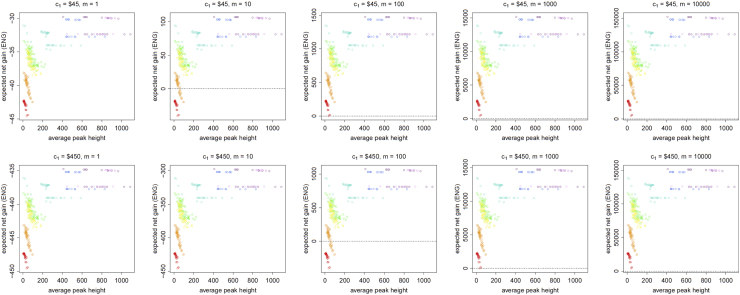
The PowerPlex 16 HS *additional replicate* data for a conservative preference structure and a probability of allele drop-in of 0.01. These graphs show the ENGs of a second replicate in function of the average allelic peak height (in rfu) of the first DNA analysis׳s EPG for DNA samples quantified as ≈0.25 pg (red), ≈0.5 pg (orange), ≈0.75 pg (yellow), ≈1 pg (green), ≈2.5 pg (turquoise), ≈5 pg (blue), ≈7.5 pg (light magenta) and ≈10 pg (dark magenta). From left to right, the graphs show the results for increasing values of the utility function׳s magnitude, *m*, for values of *m* equal to 1, 10, 100, 1000 and 10,000. The first row of graphs presents the results for a cost of $45 per DNA analysis, and the second row for a cost of $450 per DNA analysis.

**Fig. 16:**
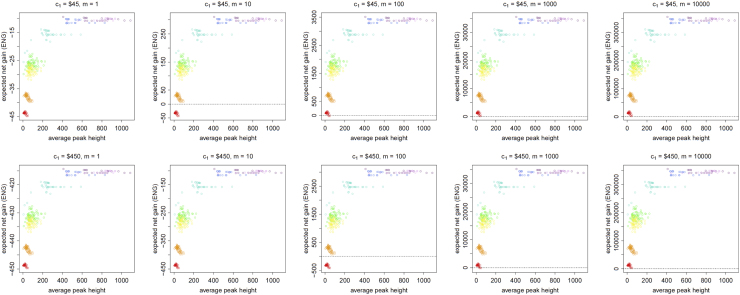
The PowerPlex 16 HS *additional replicate* data for a conservative preference structure and a probability of allele drop-in of 0.05. These graphs show the ENGs of a second replicate in function of the average allelic peak height (in rfu) of the first DNA analysis׳s EPG for DNA samples quantified as ≈0.25 pg (red), ≈0.5 pg (orange), ≈0.75 pg (yellow), ≈1 pg (green), ≈2.5 pg (turquoise), ≈5 pg (blue), ≈7.5 pg (light magenta) and ≈10 pg (dark magenta). From left to right, the graphs show the results for increasing values of the utility function׳s magnitude, *m*, for values of *m* equal to 1, 10, 100, 1000 and 10,000. The first row of graphs presents the results for a cost of $45 per DNA analysis, and the second row for a cost of $450 per DNA analysis.

**Table 1.** Logistic regression parameters for $\ln(\hat{H})$ for each kit (Identifiler Plus and PowerPlex 16 HS), donor (MT and PT) and dataset (1 and 2).

	**Identifiler Plus**	**PowerPlex 16 HS**
MT dataset 1	$\beta_{0}=6.6097$, $\beta_{1}=-1.7336$	$\beta_{0}=6.7260$, $\beta_{1}=-1.7830$
MT dataset 2	$\beta_{0}=6.1592$, $\beta_{1}=-1.6098$	$\beta_{0}=6.8470$, $\beta_{1}=-1.7725$
MT datasets 1 and 2	$\beta_{0}=6.3310$, $\beta_{1}=-1.6574$	$\beta_{0}=6.7280$, $\beta_{1}=-1.7571$
PT dataset 1	$\beta_{0}=6.5582$, $\beta_{1}=-1.7343$	$\beta_{0}=6.5978$, $\beta_{1}=-1.7495$
PT dataset 2	$\beta_{0}=6.3292$, $\beta_{1}=-1.6791$	$\beta_{0}=6.5700$, $\beta_{1}=-1.7325$
PT datasets 1 and 2	$\beta_{0}=6.3851$, $\beta_{1}=-1.6917$	$\beta_{0}=6.5981$, $\beta_{1}=-1.7429$
**All datasets**	*β*_0_=**6.3244**, *β*_1_=**−1.6632**	*β*_0_=**6.6044**, *β*_1_=**−1.7360**

## Footnotes

1 *R* version 3.1.2, freely available at http://cran.r-project.org/ (last visited April 26, 2015).
